# Seasonal accumulation of photoassimilated carbon relates to growth rate and use for new aboveground organs of young apple trees in following spring

**DOI:** 10.1093/treephys/tpac072

**Published:** 2022-07-07

**Authors:** Shogo Imada, Yasuhiro Tako

**Affiliations:** Department of Radioecology, Institute for Environmental Sciences, 1-7 Ienomae, Obuchi, Rokkasho, Kamikita, Aomori 039-3212, Japan; Department of Radioecology, Institute for Environmental Sciences, 1-7 Ienomae, Obuchi, Rokkasho, Kamikita, Aomori 039-3212, Japan

**Keywords:** ^13^CO_2_, early spring, growth season, newly formed organs, photoassimilation, woody parts

## Abstract

Deciduous trees accumulate carbon (C) in woody parts during the growth season which is subsequently used for the initial development and growth of newly formed organs in the following season; however, it is unclear which period during the growth season contributes to C accumulation. Three-year-old potted *Malus domestica* (apple) trees were grown in controlled growth chambers during the growth season and exposed to ^13^CO_2_ in an exposure chamber at seven different periods of the growth season, including vegetative and reproductive growth periods. Approximately half of the trees were harvested in late autumn, and the remaining trees were grown in a field in the following year. The ^13^C accumulation in the different organs in late autumn, and its concentration in the new aboveground growth during the following growth season, was determined. The concentration of the photoassimilated ^13^C in woody parts (shoots, trunk, rootstock and coarse roots) in the late autumn was higher in the trees labeled during the period of vigorous vegetative growth than in those labeled during other periods of growth. Furthermore, ^13^C concentration in the leaves, annual shoots, flower buds and flowers in the following early spring was also high in the trees labeled during this period. The concentration of ^13^C in the flower buds and flowers was positively correlated with that in the woody parts in the late autumn and old shoots in the following spring. Hence, the seasonal accumulation of photoassimilated C in woody parts in late autumn is related to growth rates during the growth season and its use for the initial development of newly formed organs in the following spring. These results suggest that under non-stressed conditions, C accumulated during the period of vigorous vegetative growth largely contributes to the C reserves that are used for the development of new organs in the following year.

## Introduction

Carbon (C) accumulation is an important process related to environmental tolerance, growth and long-term survival of trees (Sala et al. 2012). C photoassimilated from atmospheric CO_2_ is distributed within the plant body throughout the growth season. A fraction of the photoassimilated C is accumulated in woody parts of trees in the form of non-structural carbohydrates (NSCs), such as starch, sugars and other C compounds, which serve as stored C reserves to meet variable demands for resources under environmental stresses, such as drought, flood, wind and frost, as well as disturbances throughout the year (Palacio et al. 2014). C reserves are available in early spring for use in metabolism, development and growth of new organs, such as leaves and fine roots (Hoch et al. 2003, Sala et al. 2012). In deciduous trees, stored C is essential for the initial development and growth of newly formed organs in the following season, particularly during early spring (Hansen 1967b, Kandiah 1979). However, although trees maintain high levels of NSC in woody parts throughout the growth season (Furze et al. 2019, Hoch et al. 2003), it remains unclear which period in the growth season contributes to the allocation of assimilated C to the storage reserves, which is then available for use in the early spring.

Photoassimilated C accumulates as stored C reserves in woody parts of the trees. Although the age of stored C used in early spring varies with tree species (Kuptz et al. 2011) and forests (Gaudinski et al. 2009), it is likely that C storage pools contain a higher proportion of photoassimilated C accumulated in the previous season. Previous studies using stable (^13^C) and radioactive C isotope (^14^C) have revealed that stored C aged less than 1–2 years was used for the growth of leaf buds and fine roots in temperate deciduous forests (Gaudinski et al. 2001, 2009) and that aged 1–2 years was used for stem respiration prior to leaf development in mixed hardwood forests (Carbone et al. 2013). Richardson et al. (2015) reported that the outer tree ring (i.e., younger tissues) had young NSC pools that were not likely to be mixed with old pools, suggesting that trees would use younger stored C for growth and metabolism after dormancy. In contrast, Muhr et al. (2016) reported that relatively older stored C (three–five years old) was used for leaf growth, based on the analysis of the xylem sap of a sugar maple (*Acer saccharum*); however, it is unclear whether the trees use only stored C in the xylem sap (Gessler and Treydte 2016).

The concentration of stored C in trees fluctuates during the growth season (Furze et al. 2019, Hoch et al. 2003) and among successive years (Weber et al. 2019). Previous studies have reported that NSCs are maintained at a high level throughout the growth season and do not deplete even during periods of vigorous vegetative and reproductive growth in deciduous and evergreen trees (Furze et al. 2019, Hoch et al. 2003). Such seasonal patterns in NSC concentrations are considered to occur because the supply of photoassimilated C does not necessarily coincide with C demands for growth, respiration, and tolerance (Sala et al. 2012). Furthermore, NSC concentration in the storage organs of deciduous fruit trees decreases around bud break, reaches a minimum at the time of vigorous vegetative growth, and reaches a maximum around leaf fall (Mochizuki and Hanada 1956, Wardlaw 1990, Pallas et al. 2018, Tixier et al. 2019), although a clear trade-off relationship was not detected between NSC concentration in aboveground woody parts and trunk growth in Mediterranean tree crop species (Tixier et al. 2019). Seasonal patterns in the accumulation of C in woody parts of fruit trees can largely be influenced by changes in fruit sink strength (Wardlaw 1990, Rosati et al. 2018, Ryan et al. 2018) since fruit is a dominant sink for photoassimilates (Hansen 1967a, Monerri et al. 2011). The periods of vegetative and reproductive growth differ in fruit trees; fruit growth rate increases after shoot growth decreases in apple (*Malus domestica* Borkh) (Wardlaw 1990, Imada et al. 2017, 2021). Thus, fruit trees can be a good model for understanding the process of C accumulation in storage organs during vegetative and reproductive growth (Ryan et al. 2018).

To determine the seasonal patterns in C accumulation and its distribution during initial development in the following season, we exposed young apple trees to ^13^CO_2_ during different growth periods and determined the accumulation of photoassimilated ^13^C in woody parts in late autumn and in newly formed organs in the following growth season. Since trees generally maintain high levels of NSC in woody parts throughout the growth season (Furze et al. 2019, Hoch et al. 2003), a portion of the accumulated ^13^C in woody organs, regardless of the assimilation periods, could transfer to the newly formed organs in the following year. Based on our previous findings that allocation of photoassimilated ^13^C in apple shoots is related to growth rates (Imada et al. 2017, 2021), we hypothesized that C storage increases during the period of higher growth in woody organs.

## Materials and methods

### Study site and plant materials

This study was conducted in the field, as well as in controlled growth chambers at the Institute for Environmental Sciences (IES), Aomori, Japan (40° 57’ N, 141° 21′ E, 27 m elevation) during two successive growth seasons in 2017 and 2018. Before the experiment, 106 three-year-old *M. domestica* ‘Fuji’ trees grafted on JM.1 stocks were individually transplanted in plastic pots (25 L, 38 cm diameter, 31 cm tall) filled with a soil mixture, consisting of black soil, peat moss, vermiculite, pearlite, zeolite and soluble phosphatic manure at volume ratios of 4:2:1.2:1.2:1.2:0.5, respectively. The pH of the soil mixture was adjusted to approximately 6 by adding magnesia lime powder at a mass ratio of 0.01. The potted trees were stored in a refrigerator at 2 °C and >80% relative humidity.

### Growth conditions

All trees in the refrigerator were transported to the field in the IES on 28 April 2017 and grown until 19 May 2017, to allow for selection of trees with flower buds for the exposure experiments. A fertilizer (15-6-9 NPK) was mixed with the soil surface at 3 g N m^−2^ in each pot. The trees in the field were fully irrigated when the surface soil was dry and sprayed with pesticides, according to standard cultivation management protocols. The average dates of bud break (BBCH scale: 07, Meier 1997) and onset of leaf extension (BBCH scale: 11) were May 2 and May 8, respectively. The mean air temperature was 12 °C during the growth period in the field.

Seventy-two trees with flower buds selected based on their trunk diameter and 36 trees grown in the field for 21 days were transferred to two controlled growth chambers (5 m x 8 m x 2.3 m). The trees were aligned and placed in four rows in each growth chamber and grown until harvest. On July 24 and July 25, the trees were moved outside the facility for pesticide application. Additionally, 15-6-9 NPK fertilizer was added to the soil surface at 2 g N m^−2^ on June 21. The placement of the trees within the rows was changed at 2 or 3 week intervals and the trees were arranged in opposite directions when changing the rows to account for the non-uniformity of environmental conditions in the growth chambers. The average dates of flowering (BBCH scale: 60) and full bloom (BBCH scale: 65) were May 24 and May 25, respectively.

Air temperature of the photoperiod and dark period in the controlled growth chambers was changed at approximately ten-day intervals with reference to the values of ten-year average temperature in Aomori city (Japan Meteorological Agency) to simulate natural conditions. Meanwhile, the lengths of the photoperiod were also changed based on the sunrise and sunset times in the region, ranging between 10 and 15 h. The average air temperatures were monitored with 16 evenly distributed temperature sensors in each growth chamber; the average temperature in the photoperiod and dark period during the growth period, were 20.4 and 17.4 °C, respectively (Figure S1 available as Supplementary Data at *Tree Physiology* Online). Metal halide lamps (*n* = 78 in each chamber) (1 kW, MF1000LE/BUH, Iwasaki Electroc Co., Ltd, Tokyo, Japan) were used for lighting. The photosynthetic photon flux density was approximately 600 μmol photon m^−2^ s^−1^ from May 19 to November 5 and was measured with a quantum sensor (Apogee Instruments, Inc., Logan, USA) around the canopy height. The number of lamps was reduced to 54 in each chamber (the lighting was weakened to approximately 400 μmol photon m^−2^ s^−1^) from November 6 to 17 to decrease the air temperature. The total CO_2_ concentration in the growth chambers was usually controlled between 400 and 500 μ L^−1^ during the photoperiods.

Forty-one trees were harvested during November 13 or 17, 2017 (BBCH scale: 89). The remaining 31 trees were stored in the refrigerator after the leaves senesced. The trees were transported to the field on April 9, 2018, and randomly placed at 50–100 cm distances between the adjacent trees, and a fertilizer (6–40-6 NPK) was added to the soil surface at 6 g N m^−2^. Additionally, urea (2 g L^−1^) was sprayed on the trees on June 11 and 22, and a fertilizer (15–6-9 NPK) was added to the soil surface at 2 g N m^−2^ on August 23. The average dates of bud break (BBCH scale: 07), onset of leaf extension (BBCH scale: 11), flowering (BBCH scale: 60) and full bloom (BBCH scale: 65) of the trees were April 22, April 29, May 17 and May 21, respectively. The trees were harvested on 16 November 2018 (BBCH scale: 89).

### ^13^C labeling experiment

**Figure 1.**
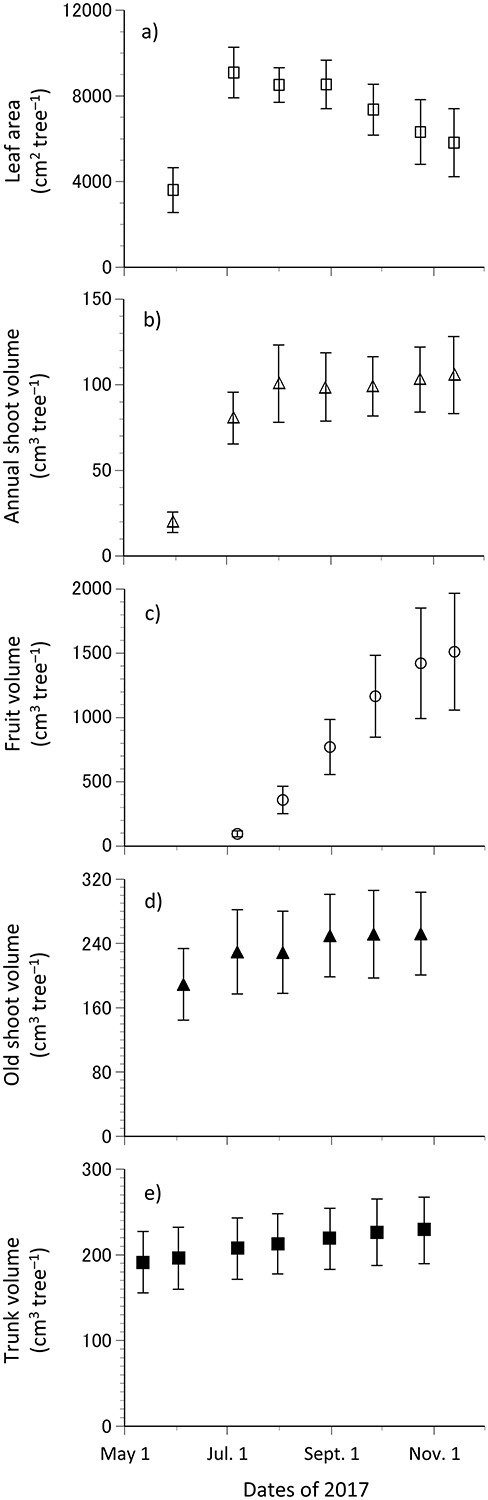
Estimated leaf area (a), volume of annual shoots (b), fruits (c), old shoots (d) and trunks of the control trees during the measurement periods in the 2017 growth season (e). Error bars denote ± SD (*n* = 5).

C isotope (^13^C) labeling was conducted by exposing the trees to ^13^CO_2_ in an exposure chamber, similar in size to the growth chamber with a function for controlling the total CO_2_ concentration and ^13^C fraction [*x* (^13^C), in %] of CO_2_ using a CO_2_-free air supplier. In addition, it also had ^13^CO_2_ cylinder, mass flow controllers, an isotope mass spectrometer (ARCO-2000, Arco System Inc., Chiba, Japan) for monitoring ^12^CO_2_ and ^13^CO_2_ concentrations with multiple sampling air selectors, and a computer system for control and data acquisition (Ohnishi Netsugaku Co., Ltd, Tokyo, Japan). The labeling experiments were performed seven times in 2017: May 31 (6 days after full bloom), June 14 (increased shoot growth, Figure 1), July 5 and July 20 (decreasing shoot growth), August 2 and August 16 (increased fruit growth) and October 11 (decreased fruit growth). Nine trees in a row were moved to the exposure chamber one day before labeling and were moved back to the growth chamber a day after labeling and grown until mid-November. The lighting system was not functional on August 2 for approximately 25 min during exposure due to system failure during the labeling experiment. Nine unlabeled trees were also grown as controls in growth chambers throughout the growth period.

The ^12^CO_2_ and ^13^CO_2_ concentrations in the exposure chamber were regulated using a computer system during the labeling experiments. The ^12^CO_2_ concentration in the exposure chamber was reduced to 340 μ L^−1^ by gas exchange until 9:00 on the days of the labeling experiment. ^13^CO_2_ gas (99%, SHOKO SCIENCE Co., Ltd, Yokohama, Japan) was intermittently injected from 9:00 onwards at a rate of 450 mL min^−1^ with a mass flow controller (ACE Inc., Kanagawa, Japan) until it reached an atmospheric *x* (^13^C) of approximately 15% (30–75 min). The ^12^CO_2_ and ^13^CO_2_ gases were added at different rates with mass flow controllers (ACE Inc., Kanagawa, Japan) until 17:00 to maintain the total ^12^CO_2_ concentration and atmospheric *x* (^13^C) at approximately 340 μ L^−1^ and 15%, respectively. Subsequently, the ^12^CO_2_ concentration was adjusted to approximately 400 μ L^−1^ from 17:00 to the end of the photoperiod. Thereafter, the air in the exposure chamber was ventilated by the outside air. The atmospheric *x* (^13^C) in the exposure chamber was confirmed to be close to its natural abundance before moving the labeled trees from the chamber. ^13^C/^12^C molar ratio of the air in the exposure chamber was determined by pumping the air out and monitoring it at three min intervals using an isotope mass spectrometer (ARCO-2000) calibrated using sensitivity calibration (N_2_ + O_2_: 21%) and reference gases (N_2_ + O_2_ + Ar + CO_2_, O_2_: 19%, Ar: 1%, CO_2_: 500–1000 ppm, and ^13^CO_2_: approximately 10 atom %) before and during the labeling experiments.

The total CO_2_ concentration and atmospheric *x* (^13^C) concentrations in the exposure chamber from 9:00 to the end of the photoperiod were similarly regulated across the different labeling days and ranged from 400 to 405 μ L^−1^ and from 0.11 to 0.13, respectively (Table S1 available as Supplementary Data at *Tree Physiology* Online). The ventilation duration (from 17:00 to the end of the photoperiod) was slightly shorter on August 16 and October 11 (3.25 and 2 h, respectively) than that on the other days (3.5 h) due to differences in the duration of the photoperiod. However, the different ventilation durations were not expected to influence the labeling conditions as the atmospheric *x* (^13^C) in the exposure chamber was decreased exponentially toward the end of the photoperiod (Table S1 available as Supplementary Data at *Tree Physiology* Online). The lighting system also turned off on August 2, albeit for a short period. Thus, the conditions of ^13^CO_2_ exposure did not differ significantly among the trees in the different labeling periods.

### Growth measurements

The sizes of all fruits, leaves, annual shoots, and old shoots of five out of the nine control trees were measured at approximately 3- to 4-week intervals during the 2017 growth season. The horizontal and vertical diameter of fruit, the length and width of leaves, the length and basal diameter of annual shoots, the diameter of old shoots with different ages, as well as the diameter of trunks were measured. The length of old shoots (of varying ages) and trunks was measured once. Leaves and annual shoots were measured during the same time. Details of the measurement analyses are available in Table S2 (available as Supplementary Data at *Tree Physiology* Online).

The volumes of fruits, annual shoots, old shoots and trunks of the control trees were estimated using the formulas of ellipsoid, cone, circular truncated cone and cylinder forms, respectively. The total leaf area was estimated from the leaf size data and the relationship between leaf size and leaf area approximated by the least square method (single leaf area = 0.672 × length × width, *n* = 303; Figure S2 available as Supplementary Data at *Tree Physiology* Online). The absolute growth rates of the estimated total leaf area (AGR_LA_), volumes of fruits (AGR_FV_), annual shoots (AGR_CBV_), old shoots (AGR_PBV_) and trunks (AGR_TV_) of the five control trees were calculated between the successive measurements of all the organs. The AGRs for the leaves, fruits and annual shoots were calculated from the time of emergence (i.e., the date of bud break or flowering). The number of annual shoots used for the calculations was 60 ± 17 (mean ± S.D., *n* = 5). The relative growth rates for trunk diameter (RGR_TD_) between the first (May 12) and last (October 26) measurements were also calculated for all the trees.

### Sampling and biomass determination

Six out of the nine control trees (including the five measurement trees) and five out of nine labeled trees (from each labeling date) were harvested on 13 November and 17 November 2017, respectively. The harvested trees were cut above the ground and separated into fruits, leaves, annual shoots, old shoots, trunk, grafting part and rootstock samples. Leaves on five long shoots (reproductive or vegetative) of each control tree (21.1 ± 6.2 cm, mean ± S.D., *n* = 25) were measured using a leaf area meter (LI-3100C; LI-COR Inc., Lincoln, NE, USA). The belowground organs were washed with tap water and divided into belowground rootstock, coarse root and fine root (≤1 mm) samples. All organs were dried at 60 °C for more than 72 h and weighed for dry mass. The data of one harvested control tree were not used and were not measured in the following analyses.

Branches of all the trees were pruned before bud break (April 12, BBCH scale: 03) in 2018. The biomass of the pruned branches was 38.4 ± 23.7 g DW tree^−1^ (mean ± S.D., *n* = 31). The pruned branch samples were divided into terminal buds and old shoots of different ages. At the pink stage (just before flowering, May 14, BBCH scale: 59), branch samples were collected and separated into leaves, annual shoots, flower buds and older branches of different ages. Flowers and fruits were collected on May 17 (flowering, BBCH scale: 60), June 6–7 (BBCH scale: 71), July 4 (BBCH scale: 74) and August 15 (BBCH scale: 77).

The trees were harvested on 16 November 2018 and separated according to the organs, as mentioned above. All plant organs were dried at 60 °C for more than 72 h and weighed for dry mass.

### Chemical analysis

Oven-dried organs were ground into a powder using a vibration sample mill or a grinder mill. The ground samples were analyzed for total C content and ^13^C/^12^C ratio using an NCH analyzer (NCH-22F, Sumika Chemical Analysis Service, Ltd, Tokyo, Japan) and mass spectrometer (DELTA V Advantage, Thermo Fisher Scientific K.K., Kanagawa, Japan), respectively. The stable C isotope composition is expressed in the δ notation (‰). Since the total C content and ^13^C/^12^C ratio were almost the same between the rootstock and belowground rootstock in 2017, the measurements were not performed for the belowground rootstock in 2018, and the values of the rootstock were used for the calculation of the belowground rootstock.

The ^13^C fraction, *x* (^13^C), was calculated using equation 1 (Dawson et al. 2002).

*x* (^13^C) = (δ^13^C/1,000 + 1) × 0.0112372/[(δ^13^C/1,000 + 1)  × 0.0112372 + 1] × 100. (1)

The *x* (^13^C) for the control trees varied among plant organs in mid-November of 2017 and 2018, as well as among new aboveground parts in 2018 (Figure S3 available as Supplementary Data at *Tree Physiology* Online), likely due to isotope discrimination (Ghashghaie and Badeck 2014). The excess atomic fraction of ^13^C [*x*^E^(^13^C), in %], and the total ^13^C recovered from plant parts, were calculated using Eqs 2 and 3, respectively.

*x*  ^E^(^13^C) = *x* (^13^C)_e_ – *x* (^13^C)_c_ (2)

Total ^13^C recovered = *x*^E^(^13^C)/100 × M (g DW), (3)

where *x* (^13^C)_e_ and *x* (^13^C)_c_ were the *x* (^13^C) in the exposed trees and the average value in the control trees, respectively. Since the *x*^E^(^13^C) of the trunk, grafting zone and rootstock were similar, the weighted average of the values was determined and presented as trunk.

The mean residence time (MRT) was estimated for flowers and fruits using the following equations:

*F*(*t*) = *a* × exp (−*k* × *d*) (4)

MRT (d) = 1/*k*, (5)

where *a*, *k* and *d* were the *x*^E^(^13^C) of flower buds (at the pink stage on May 14), the decay ratio of the *x*^E^(^13^C) and the days after the pink stage, respectively. *k* was estimated by least-squares fitting method.

### Statistical analysis

One-way analysis of variance (ANOVA) was used to determine if the RGR_TD_ in 2017, as well as the total C mass and that of woody parts in mid-November of 2017 and 2018 differed among the treatments. Differences in the AGRs of the plant organs between the different measurement periods were assessed using one-way repeated measures ANOVA using the ‘rstatix’ package. A one-way ANOVA followed by Tukey HSD multiple comparison test, or a Kruskal–Wallis test followed by Dunn’s multiple comparison test were performed using packages ‘FSA’ and ‘rcompanion,’ respectively, to determine if the *x*^E^(^13^C) and total ^13^C recovered in each organ, as well as whether MRT differed among the trees labeled in the different periods in 2017 and 2018. Data were logarithmically transformed to follow the assumptions of normality and homogeneity of variance by Shapiro–Wilk test and Levene’s test, respectively, using ‘car’ package wherever necessary. Pearson correlation analysis was conducted to evaluate the relationships among the *x*^E^(^13^C) of plant organs between 2017 and 2018, between woody parts in late November 2017 and plant organs in 2018, as well as among the *x*^E^(^13^C) of plant organs within each individual year. A linear regression analysis was conducted between woody parts in late November 2017 and one-year-old shoots in early spring (mid-April or mid-May) or flower buds in mid-May 2018 and between one-year-old shoots in early spring (mid-April or mid-May) and flower buds in mid-May 2018. The abovementioned analyses were performed using R (version 4.1.2).

## Results

### Growth and C mass

Trunk growth during the growth season and C mass (dry weight × C content) in mid-November 2017 did not vary among the control and labeled trees from different periods. The RGR_TD_ did not differ significantly (ANOVA, *P* = 0.09, *n* = 9; Figure S4 available as Supplementary Data at *Tree Physiology* Online). The C mass in the whole plants and woody parts in mid-November of 2017 (ANOVA, whole plants: *P* = 0.31, *n* = 5; woody parts: *P* = 0.50, *n* = 5) and 2018 (ANOVA, whole plants: *P* = 0.97, *n* = 3–4; woody parts: ANOVA, *P* = 0.70, *n* = 3–4; Figure S5 available as Supplementary Data at *Tree Physiology* Online) did not differ significantly among the control and labeled trees from the different periods, indicating that the experimental trees showed similar growth and C mass during the growth seasons in both the years.

The leaf area, annual shoot volume, fruit volume, old shoot volume and trunk volume were estimated at the whole-tree level for the control trees (Figure 1). The leaf area and annual shoot volume increased between late May and early July (Figure 1a and b). Meanwhile, the fruit volume increased between early August and late September (Figure 1c) and the old shoot increased between early June and early July (Figure 1d). Hence, the absolute growth rates of leaves and branches were higher during the previous growth season. Moreover, the trunk volume increased gradually during the growth season (Figure 1e). Temporal changes in their absolute growth rates were also calculated between the measurement dates during the 2017 growth season (Figure S6 available as Supplementary Data at *Tree Physiology* Online). The differences between AGR_LA_, AGR_ASV_, AGR_FV_ and AGR_OSV_ of the control trees were significant (repeated measured measures ANOVA, *P* < 0.001, *n* = 5). The AGR_TV_ was higher between mid-May and early June; however, the difference was not significant (repeated measures ANOVA, *P* = 0.26, *n* = 5).

### x^E^(^13^C) in plant organs

**Figure 2.**
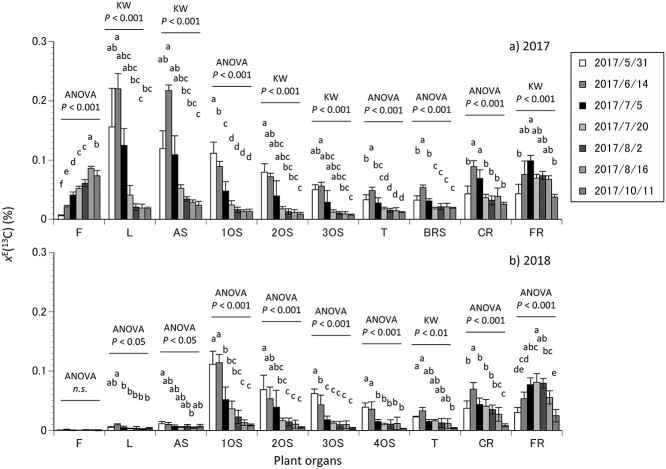
Mean *x*^E^(^13^C) of the fruits (F), leaves (L), annual shoots, 1-, 2-, 3- and 4-year-old shoots (1OS, 2OS, 3OS and 4OS), trunk (T), belowground rootstock (BRS), coarse roots (CR) and fine roots (FR) in mid-November of 2017 (a) and 2018 (b) for the trees labeled in the different periods of the 2017 growth season. One-way ANOVA followed by Tukey HSD test, or Kruskal–Wallis test followed by Dunn’s multiple comparisons test was performed for each plant organ (*P* < 0.05). The error bars denote ± SD (*n* = 5 in 2017, *n* = 4 in 2018).

The *x*^E^(^13^C) in each plant organ was calculated as a ^13^C fraction in excess of the ^13^C fraction relative to the control trees (Figure 2, Figure S3 available as Supplementary Data at *Tree Physiology* Online). The *x*^E^(^13^C) in each plant organ, during mid-November 2017 and 2018, was influenced by different periods of labeling (Figure 2). Indeed, significant variations were observed in the *x*^E^(^13^C) of each plant organ among the trees labeled in the different periods (one-way ANOVA or Kruskal–Wallis test, *P* < 0.001; Figure 2a). Moreover, the *x*^E^(^13^C) was higher in the aboveground parts (leaves, annual shoots, old shoots and trunk) in the trees labeled in late May and mid-June (one-way ANOVA or Kruskal–Wallis test, *P* < 0.001). Meanwhile, the *x*^E^(^13^C) of fruits was higher in mid-August (one-way ANOVA, *P* < 0.001). The timing of higher *x*^E^(^13^C) in the aboveground parts corresponded to the periods with higher growth rates (Figure 1). In fact, positive correlation was detected between the *x*^E^(^13^C) in leaves and woody parts (Pearson correlation, *r* = 0.77–0.88, *P* < 0.001, *n* = 35; Table S3 available as Supplementary Data at *Tree Physiology* Online). In contrast, the *x*^E^(^13^C) in the belowground rootstock and coarse roots was higher in the trees labeled in mid-June (one-way ANOVA, *P* < 0.001), similar to that in the leaves and aboveground woody parts, while that in the fine roots was higher in early July (Kruskal–Wallis test, *P* < 0.001; Figure 2a). Negative correlations were found between the *x*^E^(^13^C) in leaves (Pearson correlation, *r* = −0.80, *P* < 0.001, *n* = 35) or woody parts and fruits (Pearson correlation, *r* = −0.50, *P* < 0.01 to −0.90, *P* < 0.001, *n* = 35; Table S3 available as Supplementary Data at *Tree Physiology* Online).

Variations in the *x*^E^(^13^C) of woody parts harvested in mid-November 2018 appeared to not differ from the corresponding organs in 2017 (e.g., one-year-old branch in 2018 vs. annual shoot in 2017), although the values were lower in 2018 (Figures 2a and b). The *x*^E^(^13^C) in each woody organ in 2017 was strongly and positively correlated with that in 2018 (Pearson correlation, *r* = 0.87, *P* < 0.05 to 0.98, *P* < 0.001, *n* = 7; Table 1). In contrast, the *x*^E^(^13^C) in the fruits, leaves and annual shoots in 2018 was negligible compared to that in 2017 (Figure 2a and b), most likely due to ^13^C dilution by the current-year photoassimilates during the 2018 growth season.

**Table 1:** Pearson correlation analyses of the *x*^E^(^13^C) in each organ of the exposed trees between mid-November of 2017 and 2018

	2018											
	F	L	AS	1OS	2OS	3OS	4OS	T	GZ	RS	CR	FR
2017												
F	0.34	−0.64	−0.92^*^^*^	−0.85^*^	−0.91^*^^*^	−0.88^*^^*^	−0.75^*^	−0.77^*^	−0.82^*^	−0.67	−0.67	−0.03
L	−0.17	0.86^*^	0.89^*^^*^	0.97^*^^*^	0.94^*^^*^	0.81^*^	0.81^*^	0.92^*^^*^	0.89^*^^*^	0.83^*^	0.86^*^	0.18
AS	−0.05	0.91^*^^*^	0.83^*^	0.98^*^^*^^*^	0.89^*^^*^	0.72	0.75	0.93^*^^*^	0.91^*^^*^	0.91^*^^*^	0.93^*^^*^	0.31
1OS	−0.22	0.62	0.95^*^^*^	0.89^*^^*^	0.98^*^^*^^*^	0.97^*^^*^^*^	0.88^*^^*^	0.86^*^	0.85^*^	0.64	0.67	−0.1
2OS	−0.2	0.69	0.95^*^^*^	0.92^*^^*^	0.99^*^^*^^*^	0.96^*^^*^^*^	0.89^*^^*^	0.89^*^^*^	0.88^*^^*^	0.69	0.72	−0.03
3OS	−0.12	0.78^*^	0.94^*^^*^	0.96^*^^*^^*^	0.99^*^^*^^*^	0.91^*^^*^	0.87^*^	0.93^*^^*^	0.91^*^^*^	0.78^*^	0.81^*^	0.07
T	−0.06	0.84^*^	0.87^*^	0.98^*^^*^^*^	0.96^*^^*^^*^	0.83^*^	0.86^*^	0.96^*^^*^^*^	0.95^*^^*^	0.87^*^	0.90^*^^*^	0.24
GZ	−0.02	0.89^*^^*^	0.83^*^	0.99^*^^*^^*^	0.91^*^^*^	0.75	0.78^*^	0.95^*^^*^^*^	0.95^*^^*^	0.93^*^^*^	0.94^*^^*^	0.34
RS	0.00	0.94^*^^*^	0.81^*^	0.97^*^^*^^*^	0.86^*^	0.67	0.68	0.92^*^^*^	0.89^*^^*^	0.92^*^^*^	0.93^*^^*^	0.32
BRS	0.13	0.93^*^^*^	0.80^*^	0.95^*^^*^	0.86^*^	0.67	−0.72	0.94^*^^*^	0.88^*^^*^	0.88^*^^*^	0.91^*^^*^	0.31
CR	−0.07	0.89^*^^*^	0.58	0.82^*^	0.68	0.46	0.62	0.79^*^	0.77^*^	0.87^*^	0.89^*^^*^	0.52
FR	−0.16	0.36	−0.12	0.12	−0.02	−0.19	0.10	0.11	0.24	0.45	0.40	0.87^*^

Fruits (F), leaves (L), annual shoots (AS), 1-year-old shoots (1OS), 2-year-old shoots (2OS), 3-year-old shoots (3OS), 4-year-old shoots (4OS), trunk (T), grafting zone (GZ), rootstock (RS), belowground rootstock (BRS), coarse roots (CR), fine roots (FR).

The measurement was not performed for the BRS in 2018 (see the Materials and Methods section)

*n* = 7. ^*^  *P* < 0.05; ^*^^*^  *P* < 0.01; ^*^^*^^*^  *P* < 0.001.

### Correlation between x^E^(^13^C) in the woody parts and newly formed organs

**Figure 3.**
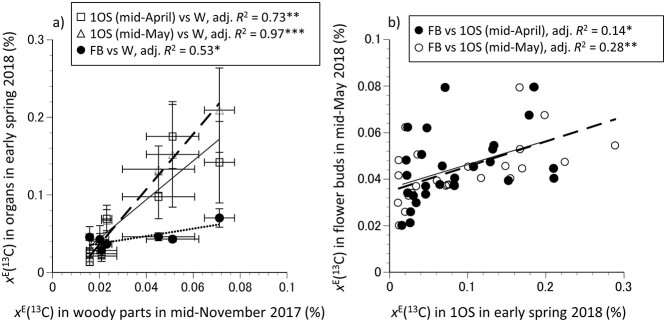
Relationship between mean *x*^E^(^13^C) in woody parts (W) in mid-November in 2017 and 1-year-old shoots (1OS) in mid-April (open square symbols), mid-May (open triangle symbols) or flower buds (FB) in mid-May in 2018 (solid circle symbols). Error bars denote ± SD (*n* = 7) (a). Relationship between mean *x*^E^(^13^C) in 1-year-old shoots (1OS) in mid-April (solid circle symbols) or mid-May in 2018 (open circle symbols) and in FB in mid-May in 2018 (*n* = 28) (b). Solid, dashed and dotted lines represent the linear regressions. ^*^  *P* < 0.05; ^*^^*^  *P* < 0.01; ^*^^*^^*^  *P* < 0.001.

Before the bud break (in mid-April) in 2018, the *x*^E^(^13^C) in the 1- and 2-year-old shoots had relatively higher values in the trees labeled in late May and mid-June (Kruskal–Wallis, *P* < 0.001; Table 2), while that in the terminal buds had higher values in the trees labeled in early July (ANOVA, *P* < 0.01). The *x*^E^(^13^C) in 1- and 2-year-old shoots was not significantly related with that in terminal buds (Table 3). At the pink stage, just before flowering (mid-May), *x*^E^(^13^C) in the leaves, annual shoots, flower buds and 1-year-old branches peaked in the trees labeled in mid-June (ANOVA or Kruskal–Wallis, *P* < 0.05, Table 2). Moreover, the *x*^E^(^13^C) in the flower buds correlated with that in the 1- (Pearson correlation, *r* = 0.41, *P* < 0.05, *n* = 28) or 2-year-old shoots in mid-April (Pearson correlation, *r* = 0.46, *P* < 0.05, *n* = 28, Table 3). Positive correlation was also observed between *x*^E^(^13^C) in woody organs in mid-November 2017 with that in 1-year-old shoots in mid-April (Pearson correlation, *r* = 0.88, *P* < 0.01, *n* = 7) and mid-May (*r* = 0.82, *P* < 0.05, *n* = 7), as well as flower buds in mid-May 2018 (*r* = 0.78, *P* < 0.05, *n* = 7), however, not with that in leaves or annual shoots (Table S4 available as Supplementary Data at *Tree Physiology* Online). Linear regression analyses further revealed that the *x*^E^(^13^C) in woody organs in late autumn 2017 was significantly associated with that in 1-year-old shoots (adjusted *R*^2^ = 0.97, *P* < 0.001, *n* = 7) and flower buds (adjusted *R*^2^ = 0.53, *P* < 0.05, *n* = 7; Figure 3a). Similarly, the *x*^E^(^13^C) in 1-year-old shoots in mid-April (adjusted *R*^2^ = 0.14, *P* < 0.05, *n* = 28) or mid-May was associated with that in flower buds (adjusted *R*^2^ = 0.28, *P* < 0.01, *n* = 28; Figure 3b).

**Table 2:** Mean *x*^E^(^13^C) in organs of the trees labeled in the different periods in 2018

Dates of samples	Dates of labeled in 2017						
	May 31	June 14	July 5	July 20	August 2	August 16	October 11
April 12, 2018 (before bud break)							
TB	0.0423 ± 0.0158^b^	0.0679 ± 0.0263^ab^	0.0951 ± 0.0295^a^	0.0830 ± 0.0158^ab^	0.0748 ± 0.0070^ab^	0.0587 ± 0.0090^ab^	0.0518 ± 0.0019^b^
1OS^†^	0.175 ± 0.041^a^	0.142 ± 0.053^ab^	0.0976 ± 0.0283^abc^	0.0691 ± 0.0176^abc^	0.0405 ± 0.0098^abc^	0.0241 ± 0.0063^bc^	0.0253 ± 0.0059^c^
2OS^†^	0.0926 ± 0.0276^a^	0.0819 ± 0.0167^a^	0.0317 ± 0.0080^ab^	0.0460 ± 0.0115^ab^	0.0246 ± 0.0110^ab^	0.0146 ± 0.0036^b^	0.0166 ± 0.0099^b^
May 14 (pink)							
L	0.0280 ± 0.0028^b^	0.0472 ± 0.0072^a^	0.0423 ± 0.0085^ab^	0.0303 ± 0.0008^ab^	0.0350 ± 0.0137^ab^	0.0263 ± 0.0076^b^	0.0370 ± 0.0081^ab^
AS	0.0422 ± 0.0055^ab^	0.0678 ± 0.0112^a^	0.0576 ± 0.0106^ab^	0.0442 ± 0.0030^ab^	0.0459 ± 0.0223^ab^	0.0400 ± 0.0084^b^	0.0477 ± 0.0106^ab^
FB^†^	0.0430 ± 0.0037^ab^	0.0703 ± 0.0119^a^	0.0461 ± 0.0051^ab^	0.0365 ± 0.0020^ab^	0.0427 ± 0.0176^ab^	0.0283 ± 0.0065^b^	0.0455 ± 0.0135^ab^
1OS^†^	0.152 ± 0.068^ab^	0.209 ± 0.054^a^	0.133 ± 0.030^abc^	0.0674 ± 0.0137^abc^	0.0361 ± 0.0091^abc^	0.0213 ± 0.0071^bc^	0.0136 ± 0.0045^c^
May 17 (flowering)							
FL	0.0432 ± 0.0071^b^	0.0682 ± 0.0132^a^	0.0446 ± 0.0055^b^	0.0329 ± 0.0097^b^	0.0369 ± 0.0128^b^	0.0257 ± 0.0077^b^	0.0347 ± 0.0071^b^
June 6 or 7							
F	0.0094 ± 0.0037^ab^	0.0132 ± 0.0119^a^	0.0076 ± 0.0051^b^	0.0055 ± 0.0020^b^	0.0075 ± 0.0176^b^	0.0077 ± 0.0076^b^	0.0088 ± 0.0081^ab^
July 4							
F^†^	0.0014 ± 0.0005	0.0017 ± 0.0041	0.0013 ± 0.0010	0.0005 ± 0.0003	0.0009 ± 0.0008	0.0008 ± 0.0006	0.0010 ± 0.0004
August 15							
F^†^	0.0004 ± 0.0005	0.0005 ± 0.0002	0.0002 ± 0.0008	0.0000 ± 0.0003	0.0000 ± 0.0005	−0.0002 ± 0.0005	0.0005 ± 0.0004

Different letters indicate significant differences among the treatments for each plant organ (*P* < 0.05, ANOVA followed by Tukey HSD multiple comparison test, or †Kruskal–Wallis test followed by Dunn’s multiple comparison test).

TB: terminal buds, FB: flower buds. See Table 1 for the other abbreviations.

**Table 3:** Pearson correlation analyses of the *x*^E^(^13^C) in plant organs on April 12, 2018 (before bud break) with those on May 14 (pink), 17 (flowering), June 6 or 7, and July 4

		May 14 (pink)				May 17 (flowering)	June 6 or 7	July 4
		L	CB	FB	1OB	FL	F	F
Apr. 12 (before bud break)								
TB	*r*	0.37	0.34	0.12	0.08	0.23	−0.06	−0.03
	*P*	0.054	0.074	0.55	0.68	0.24	0.75	0.89
1OB	*r*	0.21	0.32	0.41	0.78	0.56	0.41	0.34
	*P*	0.28	0.092	0.029	<0.001	0.0019	0.031	0.074
2OB	*r*	0.17	0.26	0.46	0.64	0.57	0.48	0.33
	*P*	0.40	0.17	0.015	<0.001	0.0014	0.011	0.084

*n* = 28

TB: terminal buds, FB: flower buds. See Table 1 for the other abbreviations.

### Total ^13^C recovered

**Figure 4.**
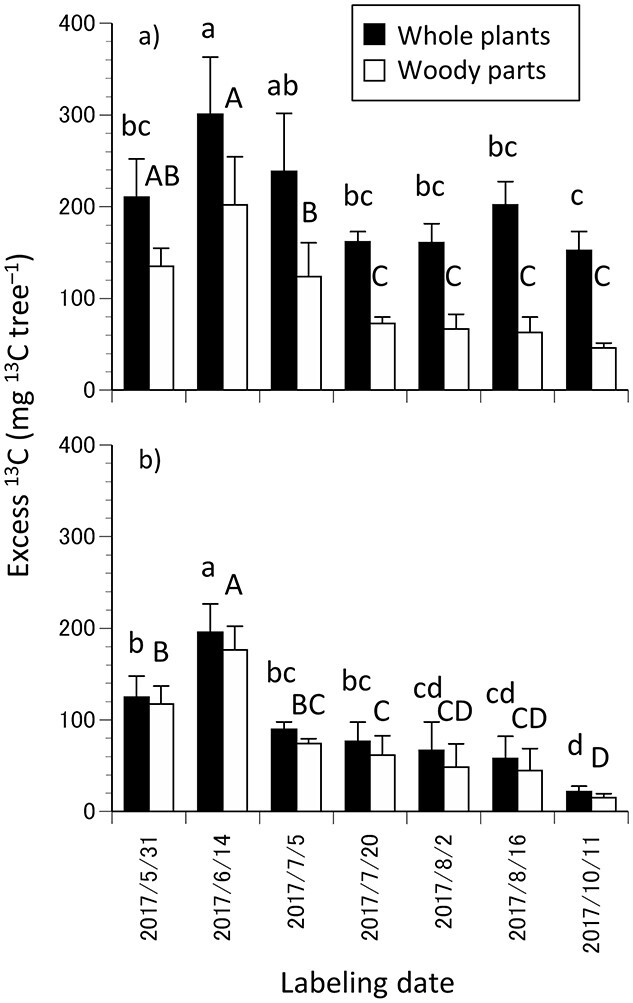
Mean excess ^13^C of total and woody parts of the trees labeled in the different periods of the growth season of 2017 in mid-November of 2017 (a) and 2018 (b). One-way ANOVA followed by Tukey HSD test was performed for the total and woody parts for each year (*P* < 0.05). The error bars denote ± SD (*n* = 5 in 2017, *n* = 4 in 2018).

The excess ^13^C in whole plants and woody parts in mid-November 2017 and 2018 was higher in the trees labeled in mid-June (ANOVA, *P* < 0.001, *n* = 5). The excess ^13^C in whole plants and woody parts was consistently lower in 2018 than that in 2017 (Figure 4). This is partly due to biomass loss caused by pruning branches before bud break in 2018 (10–28% for C mass and 17–48% for excess ^13^C in the old shoots in mid-November). Meanwhile, strong positive correlations were observed in the concentration of photoassimilated ^13^C in each woody organ between 2017 and 2018.

In 2018, the excess ^13^C in woody parts was slightly lower than that in the whole plants, unlike in 2017, due to lower excess ^13^C in the newly developed organs, particularly the fruits (Figure 4, Table S5 available as Supplementary Data at *Tree Physiology* Online). In contrast, the excess ^13^C in the fine roots was similar in 2017 and 2018 (Table S5 available as Supplementary Data at *Tree Physiology* Online).

### Temporal changes in x^E^(^13^C) in the flowers and fruits

**Figure 5.**
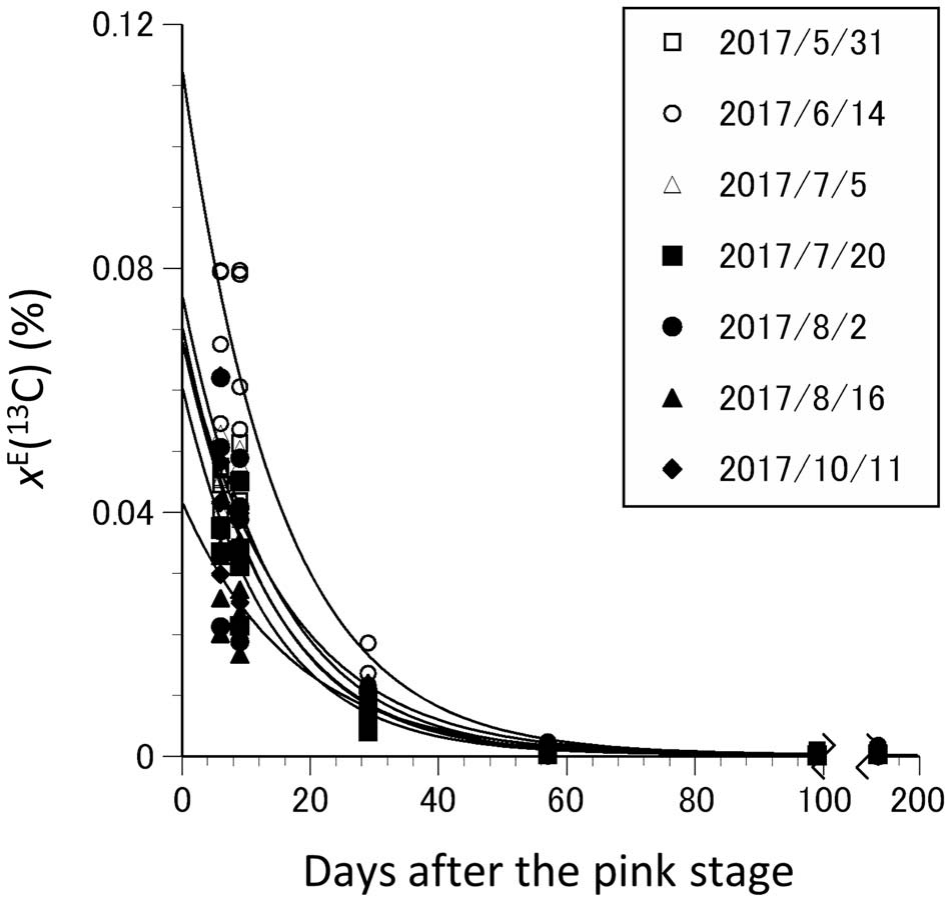
Mean remaining *x*^E^(^13^C) during the course of the growth season of 2018 after the pink stage (day 0, just before flowering, May 14) in the flower buds, flowers, and fruits of the trees labeled in the different periods of the growth season of 2017. Replicated values and average data are presented in Table 2 and Figure 3a. The *x*^E^(^13^C) values were fitted with the least square methods with a nonlinear equation [*F*(*t*) = *a* × exp (−*k* × *d*), where *a* is the *x*^E^(^13^C) at the pink stage and *k* is the decay rate of *x*^E^(^13^C)]. The flowers or fruits were fitted to *F*(*t*) = 0.0672 × exp(−0.0617 × *d*) labeled on May 31, 2017, *F*(*t*) = 0.112 × exp(−0.0665 × *d*) on June 14, *F*(*t*) = 0.0750 × exp(−0.0697 × *d*) on July 5, *F*(*t*) = 0.0599 × exp(−0.0752 × *d*) on July 20, *F*(*t*) = 0.0676 × exp(−0.0722 × *d*) on August 2, *F*(*t*) = 0.0412 × exp(−0.0576 × *d*) on August 16, and *F*(*t*) = 0.0699 × exp(−0.0743 × *d*) on October 11.

The *x*^E^(^13^C) was the highest in the flowers in mid-May, and young fruits in early June in the trees labeled in mid-June (ANOVA, *P* < 0.001 and 0.01, respectively, *n* = 4; Table 2), with patterns similar to those in the flower buds. The *x*^E^(^13^C) in the flowers and young fruits was also positively correlated with that in the 1- (Pearson correlation, *r* = 0.56, *P* < 0.01 and *r* = 0.41, *P* < 0.05, respectively, *n* = 28) or 2-year-old shoots (Pearson correlation, *r* = 0.57, *P* < 0.01 and *r* = 0.48, *P* < 0.05, respectively, *n* = 28; Table 3). The *x*^E^(^13^C) in the fruits in early July did not differ significantly among the trees labeled during different periods (Kruskal–Wallis, *P* = 0.128, *n* = 4; Table 2) and was not significantly correlated with that in the 1- (Pearson correlation, *r* = 0.34, *P* = 0.07, *n* = 28) or 2-year-old shoots before bud break (Pearson correlation, *r* = 0.33, *P* = 0.08, *n* = 28; Table 3), possibly due to the dilution via translocation of the current-year photoassimilates. The *x*^E^(^13^C) in the flower buds, flowers and fruits decreased exponentially, following an exponential decay function (Figure 5) and decreased to negligible levels in mid-August (Table 2). The average values of MRT in the trees labeled in the different periods ranged from 13.3 to 18.9 days after the pink stage (May 14). Although the initial *x*^E^(^13^C) (at the pink stage, *n* = 4) differed significantly among the trees labeled in the different periods (Kruskal–Wallis, *P* < 0.01, *n* = 4), the MRT did not differ significantly among the trees.

## Discussion

In the present study, the trees labeled in the period of vigorous vegetative growth accumulated more ^13^C in woody parts (annual shoots, old shoots, trunk, rootstock and coarse roots) by late autumn. Moreover, the concentration of photoassimilated ^13^C in newly formed organs (leaves, annual shoots, flower buds and flowers), during the early periods of the following season, was high in the trees labeled during vigorous vegetative growth. The concentration of photoassimilated ^13^C in the woody parts was positively correlated with that in the flower buds and flowers in the following early spring. These results strongly suggest that the accumulation of C, which is used in the initial development of new organs, is associated with the growth (biomass increment) rates of the woody parts during C assimilation. This may be due to a certain fraction of the photoassimilated C being allocated to a long-term storage pool (Wiley and Helliker 2012) throughout the growth season.

### Accumulation of photoassimilated C in late autumn

In late autumn, a higher concentration and accumulation of ^13^C was observed in the woody parts of the trees labeled in mid-June, when the growth rate of leaves and aboveground woody parts was higher. The increase in photoassimilated ^13^C with higher growth is primarily due to incorporation of C compounds in the form of cell wall materials, such as cellulose, hemicellulose, lignin and pectin (Murneek 1929). It has been reported that the proportion of structural carbohydrates is generally high in the leaves and branch wood of apple trees (Lareau 1988, Naschitz et al. 2010). The positive correlation between the concentration of photoassimilated ^13^C in leaves and woody parts indicates that the seasonal pattern for the accumulation of photoassimilated C was similar among the organs. Also, the periods of higher ^13^C concentration in the aboveground woody parts, particularly leaves and annual shoots, were similar to those in the belowground rootstock and coarse roots; however, they differed from those in the fine roots, suggesting that the periods of higher growth rates of the belowground rootstock and coarse roots could be similar to those of the aboveground woody parts, while differing from those of the fine roots. In general, different timings of peak growth between leaves and fine roots were observed in fruit trees, including apple (Artacho and Bonomelli 2016, Psarras et al. 2000).

The accumulation of photoassimilated ^13^C in woody parts decreased in the trees labeled in the later periods of the growth season and a negative correlation was detected between the concentration of photoassimilated ^13^C in leaves or woody parts and that in the fruits. These results indicate that the accumulation of photoassimilated ^13^C in leaves and woody parts could decrease with an increase in C accumulation in the fruits. This is consistent with the findings that with an increase in C allocation to fruits during fruit development, allocation of C decreases in the other parts of the plant (Imada et al. 2017, 2021, Rosati et al. 2018). Our results also show that photoassimilated ^13^C was detected in woody organs even in mid-August when the fruit growth rate was higher, indicating that photoassimilates were allocated to woody parts throughout the growth season, even at the time of vigorous reproductive growth. Furthermore, although isotope discrimination in plants occurs through photosynthesis (O'Leary 1981), the exposed ^13^CO_2_ concentration was considerably high, such that the effects of natural isotope discrimination on the concentration and accumulation of photoassimilated ^13^C in woody parts in late autumn are likely minimal.

### Use of photoassimilated C for initial development and growth in the following spring

The photoassimilated ^13^C from all periods of labeling was detected in new leaves, annual shoots and flower buds in the following early spring, showing that the photoassimilated C at any period of the previous growth season was incorporated into the new organs. This indicates that regardless of the assimilation period of the growth season, the photoassimilated C would be used for the initial development of new organs in the following spring. This also suggests that some of the photoassimilated C were stored in woody parts throughout the growth season, even during vigorous vegetative and reproductive growth, to be used in the following spring, which may explain why stored NSC in trees does not deplete over the year, and increases during the growth season (Furze et al. 2019, Hoch et al. 2003). However, the nature of the compounds that accumulate in the woody parts during late autumn remains unclear. Moreover, whether the compounds change according to differences in the assimilation periods is yet to be determined. According to Pollock (1986), C compounds in cell wall structures, such as fructans, may degrade and retranslocate to new organs; however, evidence regarding the role of cell wall materials as reserves in fruit trees is lacking (Loescher et al. 1990).

Similar to the concentration of photoassimilated ^13^C in woody organs in late autumn, higher concentrations of photoassimilated ^13^C were observed in the new organs in the following early spring, in the trees labeled in mid-June. The photoassimilated ^13^C in the flower buds and flowers in the following season was positively correlated with that in the woody organs in late autumn, as well as in the old shoots before bud break. These results indicate that photoassimilated C during the period of higher vegetative growth contributes more toward C storage for use in the initial growth in the following spring, although it has been shown that NSC concentration decreases in woody organs during vigorous vegetative growth (Mochizuki and Hanada 1956, Loescher et al. 1990, Hoch et al. 2003, Tixier et al. 2019). Klein et al. (2016) reported that the breakdown of starch in the branchlets of temperate deciduous tree species is the major C source for bud break and leaf development. In the present study, the concentration of photoassimilated ^13^C in the new organs correlated with that in the shoots from the previous year, but not with the terminal buds. Hence, the stored C in woody organs, particularly in old shoots, could be used for the development of new organs. Additionally, roots are also considered important sources of stored NSC for use in the development of new organs in the following spring (Loescher et al. 1990, Dovis et al. 2014); however, carbohydrate transportation from distant locations during winter and spring is not well understood (Tixier et al. 2019).

While stored C in woody parts is used for the development of new organs in the following early spring, it may not significantly contribute to subsequent growth. That is, the photoassimilated ^13^C in leaves and annual shoots during the pink stage did not correlate with that in the woody parts during late autumn, or old shoots before bud break. This is likely attributable to the differences in the onset time of the shoot and fruit growth in the trees, as the day of leaf expansion onset was 18 days earlier than that of flowering. Thus, the photoassimilated ^13^C in the leaves and annual shoots may be diluted by the translocation of current-year photoassimilates between leaf onset and flowering. Indeed, it has been reported that assimilation of CO_2_ occurs in developing leaves in early stages of leaf expansion in deciduous tree species (Keel and Schädel 2010).

The photoassimilated ^13^C in flower buds, flowers, and fruits decreased exponentially during the following growth season. The photoassimilated ^13^C reduced largely in early June (16–17 days after full bloom) and reached a negligible level in mid-August, probably because of the dilution by photoassimilate translocation from newly developing leaves and a negligible amount of redistribution of stored C from other organs. The MRT of the remaining photoassimilated ^13^C in flower buds, flowers, and fruits was estimated in our study under the assumption that C does not reallocate to other plant organs once it is incorporated into the flowers and fruits. The MRT of the remaining photoassimilated ^13^C did not vary among the trees labeled in the different periods, while the flower buds of the trees had different concentrations of photoassimilated ^13^C. This suggested that the use of photoassimilated C in the flowers and fruits would not be influenced by the assimilation periods of stored C of the previous year.

## Conclusions

The current study investigated seasonal patterns in C storage in young apple trees for use in the initial development and growth of newly developed organs in the following spring. Our results showed that the trees labeled in the period of vigorous vegetative growth (mid-June) accumulated more photoassimilated ^13^C in woody parts in late autumn, as well as in new organs during the following early spring. This supports the hypothesis that C storage, for use in the initial development and growth of new organs, increases in the woody organs during their period of higher growth rate. Our results also show that the use of stored C in the development and growth of new organs is limited to early spring. However, the trees were grown under non-stressed conditions and, therefore, the allocation patterns of photoassimilated C to storage organs for use in new organs under stressed conditions warrant further investigation.

## Authors’ Contributions

S.I. conceived and designed the research; Y.T. operated the growth and exposure chambers; S.I. and Y.T. performed the exposure experiments; S.I. performed the data collection, analyzed the data, and wrote the manuscript; both authors have read the manuscript and commented on it.

## Supplementary Material

supporting_Information_revisioin_soac086
