# Supplementary material for: Seasonal accumulation of photoassimilated carbon relates to growth rate and use for new aboveground organs of young apple trees in following spring
Source: Tree Physiol. 2022 Jul 7;42(11):2294–305. doi: 10.1093/treephys/tpac072 (PMC9652006; doi:10.1093/treephys/tpac072)
Supplement: supporting_Information_revisioin_soac086 [file supporting_information_revisioin_soac086.docx]

## Supplementary data

Article title: Seasonal accumulation of photoassimilated carbon relates to growth rate and use for new organs of young apple trees in following spring

Authors: Shogo Imada, Yasuhiro Tako

The following Supplementary Data is available for this article:

Figure S1. Mean air temperature in the light and dark period in the two controlled growth chambers during the 2017 growth season.

Figure S2. Relationship between leaf area and the product of leaf length and width of the control trees collected in mid-November 2017.

Figure S3. Mean ^13^C fraction of CO_2_ [*x* (^13^C)] in plant organs of the control trees in mid-November 2017 and 2018 (a) and during the growth season of 2018 (b).

Figure S4. Mean relative growth rate of trunk diameter of the control trees and the trees labeled during the different periods between mid-May to late-October 2017.

Figure S5. Mean C mass of woody parts and whole plants of the control and exposed trees in mid-November of 2017 (a) and 2018 (b).

Figure S6. Mean absolute growth rates of estimated leaf area (AGR_LA_) (a). Volumes of annual shoot (AGR_ASV_) (b), fruit (AGR_FV_) (c), old shoot (AGR_OSV_) (d), and trunk (AGR_TV_) (e) of the control trees between the measurement periods during the 2017 growth season.

Table S1. Dates of exposure experiments, average air temperature, total CO_2_ concentration, *x* (^13^C) of CO_2_, exposure time, and days until harvest.

Table S2. Plant organs, measurement items, and measurement dates during the growth season of 2017.

Table S3. Pearson correlation analyses of the *x*^E^(^13^C) among the organs of the exposed trees in 2017 and 2018.

Table S4. Pearson correlation analyses of the mean *x*^E^(^13^C) in woody parts on November 17, 2017 with that in plant organs on April 12 (before bud break), May 14 (pink), May 17 (flowering), June 6 or 7, and July 4.

Table S5. Mean excess ^13^C in the organs of the trees labeled in the different periods in mid-November of 2017 and 2018.

Figure S1. Mean air temperature in the light and dark period in the two controlled growth chambers during the 2017 growth season. The error bars denote ± SD (n = 31–32).

Figure S2. Relationship between leaf area and the product of leaf length and width of the control trees collected in mid-November 2017. Leaf area = 0.672 × leaf length × leaf width (*r*^2^ = 0.96, n = 303).

Figure S3. Mean ^13^C fraction of CO_2_ [*x* (^13^C)] in plant organs of the control trees in mid-November 2017 and 2018 (a) and during the growth season of 2018 (b). The error bars denote ± SD (n = 5 in 2017, n = 3 in 2018). The trees were grown in the controlled growth chambers from late-May to mid-November 2017 and in the field throughout the growth season of 2018. See Tables 1 and 3 for the abbreviations.

Figure S4. Mean relative growth rate of trunk diameter of the control trees and the trees labeled during the different periods between mid-May to late-October 2017. The error bars denote ± SD (n = 9)

Figure S5. Mean C mass of woody parts and whole plants of the control and exposed trees in mid-November of 2017 (a) and 2018 (b). One-way ANOVA was performed for the total and woody parts of each year. The error bars denote ± SD (n = 5 in 2017, n = 3–4 in 2018).

Figure S6. Mean absolute growth rates of estimated leaf area (AGR_LA_) (a). Volumes of annual shoot (AGR_ASV_) (b), fruit (AGR_FV_) (c), old shoot (AGR_OSV_) (d), and trunk (AGR_TV_) (e) of the control trees between the measurement periods during the 2017 growth season. A one-way repeated measures ANOVA was performed for each plant organ (*P* <0.05). Error bars denote ± SD (n = 5).

Table S1. Date of exposure experiments, average air temperature, total CO^2^ concentration, *x* (^13^C) of CO_2_ during exposure experiment (9:00–end of photoperiod), ^13^CO_2_ injection (9:00–17:00), and ventilation by outside air (17:00–end of photoperiod), exposure time, and days until harvest (November 17 2017)

| Labeling dates | Time | Air temperature | Total CO_2_ | ^13^C/(^13^C + ^12^C) | Exposure time | Days until harvest |
| --- | --- | --- | --- | --- | --- | --- |
|  |  | (°C) | (μL l^–1^) | molar ratio | (h) | (d) |
| May 31, 2017 | 9:00–20:30 | 18.3 | 400 | 0.11 | 11.50 | 170 |
|  | 9:00–17:00 | 18.1 | 391 | 0.14 |  |  |
|  | 17:00–20:30 | 18.8 | 420 | 0.057 |  |  |
| Jun. 14 | 9:00–20:30 | 17.6 | 400 | 0.11 | 11.50 | 156 |
|  | 9:00–17:00 | 17.5 | 391 | 0.13 |  |  |
|  | 17:00–20:30 | 17.9 | 421 | 0.065 |  |  |
| Jul. 5 | 9:00–20:30 | 20.0 | 401 | 0.12 | 11.50 | 135 |
|  | 9:00–17:00 | 19.9 | 393 | 0.14 |  |  |
|  | 17:00–20:30 | 20.2 | 417 | 0.059 |  |  |
| Jul. 20 | 9:00–20:30 | 22.3 | 402 | 0.12 | 11.50 | 120 |
|  | 9:00–17:00 | 22.3 | 395 | 0.15 |  |  |
|  | 17:00–20:30 | 22.3 | 420 | 0.063 |  |  |
| Aug. 2 | 9:00–20:30 | 23.4 | 405 | 0.12 | 11.05^*^ | 107 |
|  | 9:00–17:00 | 23.4 | 398 | 0.15 |  |  |
|  | 17:00–20:30 | 23.5 | 420 | 0.061 |  |  |
| Aug. 16 | 9:00–20:15 | 24.2 | 404 | 0.12 | 11.25 | 93 |
|  | 9:00–17:00 | 24.2 | 396 | 0.15 |  |  |
|  | 17:00–20:15 | 24.2 | 423 | 0.063 |  |  |
| Oct. 11 | 9:00–19:00 | 19.3 | 403 | 0.13 | 10.00 | 37 |
|  | 9:00–17:00 | 19.2 | 396 | 0.14 |  |  |
|  | 17:00–19:00 | 19.4 | 430 | 0.088 |  |  |

Data for ^12^CO_2_ and ^13^CO_2_ were collected 3 min intervals during exposure experiments

^*^The time of lights-out (*c*. 25 min) due to system failure was excluded.

Table S2. Plant organs, measurement items, and measurement dates during the growth season of 2017.

| Plant parts | Measurement items | Measurement date |
| --- | --- | --- |
| Fruits | Horizontal (two orthogonal directions) and vertical diameters | July 7, Aug 3, Aug 31, Sep 27, Oct 24 to 25, and Nov 13 |
| Leaves | Length and width of lamina | May 30 to June 1, July 4 to 6, Aug 1 to 3, Aug 29 to 30, Sep 26 to 27, and Oct 24 to 25 |
| Annual shoots | Length and basal diameter (two orthogonal directions) |  |
| old shoots | Tip and basal diameters (two orthogonal directions, respectively) | June 5, July 7, Aug 3, Aug 31, Sep 26 to 27, and Oct 24 to 25 |
|  | Length | June 5 |
| Trunk | Diameter (two orthogonal directions) at the height of 5 cm from the soil surface | May 12, June 2, July 7, July 31, Aug 31, Sept 28, and Oct 26 |
|  | Length | Nov 13 |

Table S3. Pearson correlation analyses of the *x*^E^(^13^C) among the organs of the exposed trees in 2017 and 2018.

| 1. 2017 |  | | |  | |  | |  | |  | |  |  |  | |  | |  | |  | |
| --- | --- | --- | --- | --- | --- | --- | --- | --- | --- | --- | --- | --- | --- | --- | --- | --- | --- | --- | --- | --- | --- |
|  | F | | | L | | CB | | 1OB | | 2OB | | 3OB | T | GZ | | RS | | CR | | FR | |
| L | –0.80*** | | |  | |  | |  | |  | |  |  |  | |  | |  | |  | |
| CB | –0.76*** | | | 0.88*** | |  | |  | |  | |  |  |  | |  | |  | |  | |
| 1OB | –0.90*** | | | 0.84*** | | 0.81*** | |  | |  | |  |  |  | |  | |  | |  | |
| 2OB | –0.85*** | | | 0.86*** | | 0.85*** | | 0.96*** | |  | |  |  |  | |  | |  | |  | |
| 3OB | –0.82*** | | | 0.87*** | | 0.92*** | | 0.93*** | | 0.97*** | |  |  |  | |  | |  | |  | |
| T | –0.76*** | | | 0.83*** | | 0.93*** | | 0.87*** | | 0.91*** | | 0.95*** |  |  | |  | |  | |  | |
| GZ | –0.71*** | | | 0.84*** | | 0.90*** | | 0.79*** | | 0.88*** | | 0.89*** | 0.90*** |  | |  | |  | |  | |
| RS | –0.68*** | | | 0.81*** | | 0.91*** | | 0.75*** | | 0.78*** | | 0.83*** | 0.89*** | 0.91*** | |  | |  | |  | |
| BRS | –0.64*** | | | 0.85*** | | 0.90*** | | 0.73*** | | 0.79*** | | 0.83*** | 0.87*** | 0.90*** | | 0.91*** | |  | |  | |
| CR | –0.50** | | | 0.77*** | | 0.85*** | | 0.59*** | | 0.65*** | | 0.74*** | 0.83*** | 0.80*** | | 0.83*** | | 0.87*** | |  | |
| FR | –0.03 | | | 0.14 | | 0.24 | | –0.06 | | –0.01 | | 0.05 | 0.19 | 0.2 | | 0.21 | | 0.22 | | 0.53** | |
| 1. 2018 | |  |  | |  | |  | |  | |  | |  | |  | |  | |  | |  |
|  | | F | L | | CB | | 1OB | | 2OB | | 3OB | | 4OB | | T | | GZ | | RS | | CR |
| L | | 0.26 |  | |  | |  | |  | |  | |  | |  | |  | |  | |  |
| CB | | 0.10 | 0.70*** | |  | |  | |  | |  | |  | |  | |  | |  | |  |
| 1OB | | –0.05 | 0.71*** | | 0.63*** | |  | |  | |  | |  | |  | |  | |  | |  |
| 2OB | | 0.10 | 0.62*** | | 0.62*** | | 0.78*** | |  | |  | |  | |  | |  | |  | |  |
| 3OB | | –0.05 | 0.56** | | 0.60*** | | 0.91*** | | 0.85*** | |  | |  | |  | |  | |  | |  |
| 4OB | | 0.06 | 0.64*** | | 0.67*** | | 0.89*** | | 0.83*** | | 0.92*** | |  | |  | |  | |  | |  |
| T | | 0.13 | 0.67*** | | 0.51** | | 0.85*** | | 0.76*** | | 0.87*** | | 0.93*** | |  | |  | |  | |  |
| GZ | | –0.02 | 0.54** | | 0.39* | | 0.74*** | | 0.56** | | 0.66*** | | 0.77*** | | 0.82*** | |  | |  | |  |
| RS | | 0.16 | 0.68*** | | 0.37 | | 0.70*** | | 0.51** | | 0.51** | | 0.63*** | | 0.75*** | | 0.81*** | |  | |  |
| CR | | 0.14 | 0.58** | | 0.28 | | 0.67*** | | 0.57** | | 0.51** | | 0.66*** | | 0.76*** | | 0.83*** | | 0.90*** | |  |
| FR | | 0.07 | –0.12 | | –0.24 | | –0.17 | | –0.18 | | –0.32 | | –0.20 | | –0.12 | | 0.21 | | 0.30 | | 0.40* |

*n* = 35 in 2017, *n* = 28 in 2018. * *P* < 0.05; ** *P* < 0.01; *** *P* < 0.001.

See Table 1 for the abbreviations.

Table S4. Pearson correlation analyses of the mean *x*^E^(^13^C) in woody parts on November 17, 2017 with that in plant organs on April 12 (before bud break), May 14 (pink), May 17 (flowering), June 6 or 7, and July 4.

|  | April 12, 2018 | | |  | May 14 | | | |  | May 17 |  | June 6 or 7 |  | July 4 |
| --- | --- | --- | --- | --- | --- | --- | --- | --- | --- | --- | --- | --- | --- | --- |
|  | TB | 1OS | 2OS |  | L | AS | FB | 1OS |  | FL |  | F |  | F |
| Woody parts in  November 17, 2017 | 0.0087 | 0.88** | 0.82* |  | 0.56 | 0.74 | 0.78* | 0.99*** |  | 0.91** |  | 0.77* |  | 0.89** |

*n* = 7, * *P* < 0.05; ** *P* < 0.01; *** *P* < 0.001.

See Tables 1 and 2 for the abbreviations.

Table S5. Mean excess ^13^C in the organs of the trees labeled in the different periods in mid-November of 2017 and 2018.

| Organ | Excess ^13^C (mg ^13^C) | | | | | | |
| --- | --- | --- | --- | --- | --- | --- | --- |
|  | May 31, 2017 | June 14 | July 5 | July 20 | August 2 | August 16 | October 11 |
| In mid-November of 2017 | | | | | | | |
| F | 8.1 ± 1.7^f^ | 22.5 ± 2.9^e^ | 50.7 ± 9.9^d^ | 61.5 ± 4.9^cd^ | 74.8 ± 15.7^bc^ | 122.4 ± 11.5^a^ | 94.8 ± 17.4^ab^ |
| L^†^ | 60.2 ± 32.5^a^ | 62.5 ± 14.3^a^ | 46.8 ± 22.7^ab^ | 16.0 ± 9.4^ab^ | 6.8 ± 2.9^ab^ | 5.7 ± 2.4^b^ | 5.1 ± 1.3^b^ |
| CB | 25.4 ± 5.8^a^ | 36.6 ± 9.4^a^ | 26.6 ± 15.6^a^ | 11.8 ± 1.4^b^ | 7.3 ± 2.4^bc^ | 6.6 ± 1.3^bc^ | 4.9 ± 1.8^c^ |
| 1–3OB | 43.8 ± 12.0^a^ | 47.0 ± 13.1^a^ | 25.6 ± 10.6^ab^ | 13.4 ± 3.2^bc^ | 11.1 ± 4.7^c^ | 7.5 ± 2.7^c^ | 6.6 ± 2.3^c^ |
| T | 34.0 ± 5.2^ab^ | 51.6 ± 13.8^a^ | 27.7 ± 7.1^bc^ | 19.1 ± 1.8^cd^ | 17.7 ± 3.0^def^ | 16.1 ± 6.3^ef^ | 11.8 ± 1.1^f^ |
| BRS | 14.0± 3.7^b^ | 25.7 ± 6.7^a^ | 11.9 ± 3.4^b^ | 9.6 ± 2.9^b^ | 9.9 ± 3.9^b^ | 9.1 ± 2.8^b^ | 8.5 ± 1.9^ab^ |
| CR | 18.0 ± 6.9^bc^ | 41.2 ± 15.6^a^ | 32.0 ± 6.5^ab^ | 18.9 ± 2.9^bc^ | 20.7 ± 5.5^bc^ | 23.8 ± 7.1^bc^ | 14.4 ± 3.5^c^ |
| FR | 7.3 ± 2.8^bc^ | 13.9 ± 6.3^ab^ | 17.4 ± 4.8^a^ | 11.7 ± 3.1^abc^ | 12.5 ± 3.3^ab^ | 10.8 ± 1.4^abc^ | 6.4 ± 1.9^c^ |
| In mid-November of 2018 | | | | | | | |
| F | 0.0 ± 2.1 | 1.2 ± 1.6 | –0.6 ± 2.1 | 0.0 ± 0.6 | 1.3 ± 1.0 | 1.1 ± 0.8 | 0.6 ± 1.1 |
| L | 0.8 ± 0.3^ab^ | 1.8 ± 0.3^a^ | 1.1 ± 0.8^ab^ | 0.5 ± 0.2^b^ | 0.7 ± 0.6^b^ | 0.3 ± 0.2^b^ | 0.6 ± 0.3^b^ |
| CB | 1.5 ± 0.6^a^ | 1.4 ± 0.6^ab^ | 1.0 ± 0.4^ab^ | 0.7 ± 0.3^ab^ | 0.9 ± 0.3^ab^ | 0.6 ± 0.2^b^ | 0.8 ± 0.3^ab^ |
| 1–4OB | 61.0 ± 11.3^a^ | 69.1 ± 6.0^a^ | 26.8 ± 6.4^b^ | 15.7 ± 5.1^bc^ | 10.9 ± 7.4^bc^ | 12.4 ± 7.4^bc^ | 3.8 ± 1.1^c^ |
| T | 25.8 ± 3.0^ab^ | 41.2 ± 5.0^a^ | 17.4 ± 2.0^b^ | 17.5 ± 5.7^b^ | 14.0 ± 9.0^b^ | 13.1 ± 8.4^b^ | 4.2 ± 1.4^c^ |
| BRS | 9.7 ± 2.9^ab^ | 21.8 ± 9.0^a^ | 10.2 ± 1.9^ab^ | 8.8 ± 3.1^ab^ | 8.9 ± 4.7^b^ | 6.3 ± 3.0^bc^ | 2.8 ± 0.9^c^ |
| CR | 19.5 ± 10.5^ab^ | 43.1 ± 15.3^a^ | 19.0 ± 3.1^ab^ | 19.1 ± 9.5^ab^ | 13.8 ± 5.6^b^ | 12.5 ± 5.5^b^ | 3.8 ± 2.1^c^ |
| FR | 6.5 ± 1.9^bc^ | 16.3 ± 5.3^a^ | 14.8 ± 4.8^ab^ | 14.1 ± 1.4^ab^ | 16.1 ± 5.2^a^ | 11.8 ± 2.8^abc^ | 5.4 ± 2.0^c^ |

Different letters indicate significant differences among the treatments for each plant organ (*P* < 0.05, ANOVA followed by Tukey HSD multiple comparison test or ^†^Kruskal–Wallis test followed by Dunn's multiple comparison test)

See Table 1 for the abbreviations.
